# Theoretical Study of the Phonon Energy and Specific Heat of Ion-Doped LiCsSO_4_—Bulk and Nanoparticles

**DOI:** 10.3390/ma17122845

**Published:** 2024-06-11

**Authors:** Angel T. Apostolov, Iliana N. Apostolova, Julia Mihailowa Wesselinowa

**Affiliations:** 1 Civil Engineering and Geodesy, University of Architecture, Hr. Smirnenski Blvd. 1, 1046 Sofia, Bulgaria; angelapos@abv.bg; 2 University of Forestry, Kl. Ohridsky Blvd. 10, 1756 Sofia, Bulgaria; inaapos@abv.bg; 3 Faculty of Physics, Sofia University “St. Kliment Ohridski”, J. Bouchier Blvd. 5, 1164 Sofia, Bulgaria

**Keywords:** LiCsSO_4_, ion doping, phonon energy, phase transition temperature, specific heat, microscopic model

## Abstract

Using a microscopic model, the temperature dependence of two phonon modes, $\omega_{0}$ = 32 cm^−1^ and 72 cm^−1^, and their damping of the ferroelastic LiCsSO_4_ compound, are calculated within Green’s function technique. It is observed that the first mode increases whereas the second one decreases with increasing temperature *T*. This different behavior is explained with different sign of the anharmonic spin–phonon interaction constant. At the ferroelastic phase transition temperature $T_{C}$, there is a kink in both modes due to the spin–phonon interaction. The phonon damping increases with *T*, and again shows an anomaly at $T_{C}$. The contributions of the spin–phonon and phonon–phonon interactions are discussed. $T_{C}$ is reduced by decreasing the nanoparticle size, and can be enhanced by doping with K, Rb and NH_4_ ions at the Cs site. $T_{C}$ decreases by doping with Na, K or Rb on the Li site. The specific heat $C_{p}$ also shows a kink at $T_{C}$. $C_{p}$ decreases with decreasing nanoparticle size and the peak disappears, whereas $C_{p}$ increases with increasing K ion doping concentration.

## 1. Introduction

Ferroelasticity is a phenomenon where a material demonstrates spontaneous strain. In the realm of ferroics, ferroelasticity serves as the mechanical analog to ferroelectricity and ferromagnetism. When stress is applied to a ferroelastic material, it transitions from one stable phase to another equally stable phase, which may involve a change in the crystal structure (such as cubic to tetragonal) or a different orientation (a ’twin’ phase). This stress-induced phase transition leads to spontaneous strain in the material. LiCsSO_4_ (LCS) is of interest as a material undergoing a phase transformation and having ferroelastic properties at low temperatures [1]. At room temperature, LCS crystals exhibit an orthorhombic pseudo-hexagonal symmetry and belong to the space group Pcmn. LCS undergoes a second-order structural phase transition at $T_{C}$∼202 K, shifting from the paraelastic phase to the ferroelastic monoclinic structure without altering the unit cell content [2,3,4]. This transition is of the order–disorder type. The mechanism driving the ordered phase involves rotations of the SO_4_ tetrahedra within the ab plane [3,5]. But, the transition mechanism in LCS from the para- to the ferroelastic phase remains unclear.

The ferroealastic phase transition $T_{C}$ is theoretically described within the hcp Ising model [6,7,8,9,10]. Hasebe and Asahi [11] have discussed the phase transition of LCS by the order parameter–shear strain $x_{6}$ coupled model. The soft mode theory is used by Zhou et al. [12] to study the $T_{C}$ of the LCS crystal. Tuszynski et al. [13] made comments on the hysteresis loop in ferroelastic LCS using the Landau-based free-energy expansion.

The Raman lattice modes $A_{g}$ and $B_{g}$ in LCS crystals were examined across a temperature span of 17–303 K [14,15,16,17]. Raman spectra for LCS were recorded with polarization aligned to $A_{g}$ symmetry and with crossed polarization corresponding to the B_1*g*_, B_2*g*_, and B_3*g*_ symmetries. The Raman lines in LCS are categorized into three distinct frequency regions: 0 to 200 cm^−1^, 360 to 660 cm^−1^, and 1000 to 1200 cm^−1^ [15]. The lowest frequency range encompasses the translational vibrations of Li^+^ ions, while the intermediate frequency range is attributed to the librational motions of sulfate ions. The highest frequency range includes bands identified as modes derived from the stretching vibrations, specifically $\nu_{1}$ at ω = 1016 cm^−1^, and $\nu_{3}$, at frequencies between 1110 and 1200 cm^−1^. The behavior of the surface phonons in the vicinity of the phase transition temperature $T_{C}$ was studied by Trzaskowska et al. [18,19] using Brillouin spectroscopy. Recently, the size effects of the linear permittivity $\epsilon^{\prime }$ in ferroelastic LCS nanoparticles (NPs) were investigated by Milinskiy et al. [20]. The measurements were carried out by linear and non-linear methods of dielectric spectroscopy. The phase transition temperature $T_{C}$ is reduced compared to that in the bulk LCS, as reported by Borisov et al. [21].

Ion doping effects with different ions, such as Rb, NH_4_, Cu, Mn, etc., on the phase transition temperature $T_{C}$ in the bulk LCS are reported by Czaja [22], Zapart et al. [23], Lima et al. [24], and Misra et al. [25]. They observed tuning of the $T_{C}$. It is expected that, as a result of substituting Cs^+^ ions with other ions, a modification of the ferroic properties will take place. A strong increase in the $T_{C}$ is reported by Czaja et al. [22] for the NH_4_-doped LCS, from 202 to 230.8 K for the doping concentration *x* = 0.15. Zapart et al. [23] determined that, in Rb-doped LCS, the phase transition temperature is $T_{C}$ = 215 K, i.e., 13 K above that in pure LCS. Lima et al. [24] have investigated temperature-dependent Raman scattering studies in Rb-doped LCS for *x* = 0.35 in the temperature range of 7-295 K. They have shown that the doped compound undergoes a phase transition at a $T_{C}$ of about 275 K. Misra et al. [25] have performed EPR studies on Mn^+^-doped LCS in the temperature range of 3.8–301 K, as well as on Cu^+^-doped LCS at room temperature.

The aim of the present paper is to theoretically study the phonon properties of ferroelastic LCS, as well as the size and ion doping effects on the phase transition temperature $T_{C}$ and the specific heat $C_{p}$ which, to our knowledge, has not been performed until now.

## 2. Model and Method

The Hamiltonian that describes the properties of ferroelastic LCS is the spin-1/2 hcp Ising model [6]:

(1) $$H=-\frac{1}{2}\sum_{ij}J_{ij}S_{i}S_{j}.$$

Here, $S_{i}$ represents the pseudo-spin operator at site *i*. The exchange interaction $J_{ij}$ = $J_{1}$ corresponds to nearest neighbor pairs within the hcp (ab) plane, while $J_{ij}$ = $J_{2}$ pertains to nearest neighbor pairs outside of the plane [6]. It is postulated that $J_{1}$ may exhibit either ferromagnetic or antiferromagnetic characteristics, whereas the second exchange integral $J_{2}$ remains ferromagnetic, $|J_{1}|>J_{2}$, $J\left(q\right)=J_{1}+J_{2}\cos\left(bq/3\right)$.

To include the spin dynamics in the Ising model, we additively take into account the spin–phonon and phonon–phonon interaction terms, $H_{sp-ph}$ and $H_{ph-ph}$:

(2) $$H_{sp-ph}=-\sum_{k,q,p}R\left(k,q,p\right)S_{q}S_{-p}Q_{k}Q_{p-q-k},$$

where $Q_{i}$ is the normal coordinate, and can be expressed in terms of phonon creation $a^{+}$ and annihilation *a* operators: $Q_{i}={\left(2\omega_{0i}\right)}^{-1/2}\left(a_{i}+a_{i}^{+}\right)$. *R* is the anharmonic spin–phonon interaction constant. $\omega_{0i}$ is the frequency of the lattice mode.

$H_{ph-ph}$ describes the lattice vibrations, including anharmonic phonon–phonon interactions:

(3) $$\begin{array}{ccc} H_{ph-ph} & = & \frac{1}{2!}\sum_{i}\omega_{0i}a_{i}a_{i}^{+}+\frac{1}{3!}\sum_{i,j,r}B\left(i,j,r\right)Q_{i}Q_{j}Q_{r}\\ & + & \frac{1}{4!}\sum_{i,j,r,s}A\left(i,j,r,s\right)Q_{i}Q_{j}Q_{r}Q_{s}, \end{array}$$

where *A* and *B* are the three-phonon and four-phonon anharmonic interaction constants, respectively.

From the phonon’s Green function, using the method of Tserkovnikov [26], which allows us to also calculate the damping effects,

(4) $${\bar{G}}_{ij}\left(t\right)=\langle \langle a_{i}\left(t\right);a_{j}^{+}\rangle \rangle$$

we observed the phonon energies and damping.

## 3. Numerical Results and Discussion

Numerical calculations were executed within the JAVA programming environment, employing straightforward iterative methodologies and summation over closest neighboring entities. Utilizing the specified model parameters, the properties of LCS are computed: $|J_{2}/J_{1}|$ = 0.3 [10], $T_{C}$ = 202 K, ∣R∣ = −20 cm^−1^, *A* = 6.7 cm^−1^, *B* = −3.1 cm^−1^.

### 3.1. Temperature Dependence of the A_*1*g_ Phonon Modes $\omega_{0}$ = 32 and 72 cm*^−1^* in Bulk LCS

**Figure 1:**
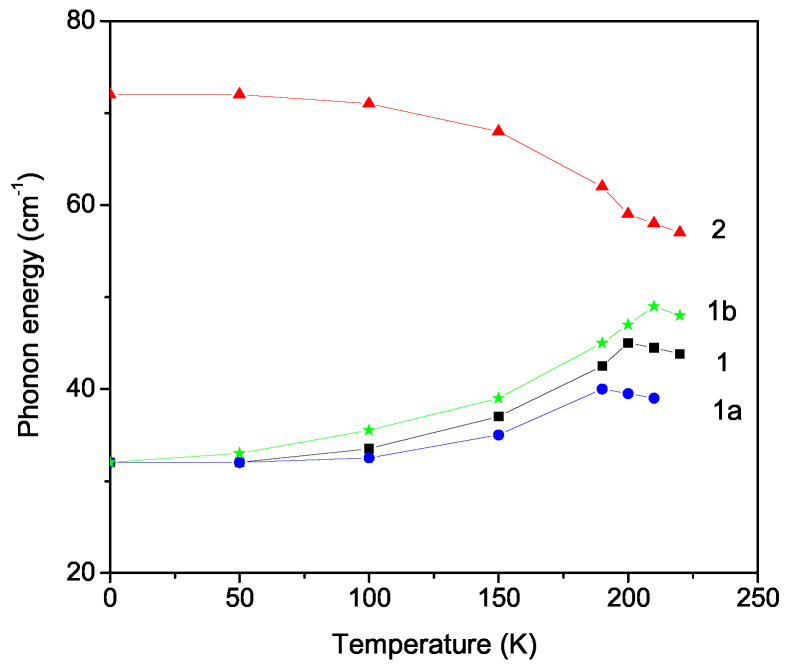
Temperature dependence of the phonon energy in bulk LCS for two modes $\omega_{0}$ = 32 cm^−1^, R>0 (1), and 72 cm^−1^, R<0 (2), with $|J_{2}/J_{1}|$ = 0.3, and for $\omega_{0}$ = 32 cm^−1^ with $|J_{2}/J_{1}|$ = 0.15 (1a) and 0.5 (1b).

The temperature dependence of the phonon energies of the A_1*g*_ modes $\omega_{0}$ = 32 and 72 cm^−1^ were evaluated. They are connected with the translational Li^+^ vibrations. Let us note that we can also investigate the other phonon modes within our model and method. The results are shown in Figure 1. It can be seen that the phonon energy for the $\omega_{0}$ = 0.32 cm^−1^ mode increases with an increase in temperature *T* (curve 1), whereas for the other mode ω = 72 cm^−1^, it decreases with *T* (curve 2). In order to explain this different temperature behavior, for the first case, we must chose a positive anharmonic spin–phonon interaction constant, R>0 (curve 1), and a positive one for the second case, R<0 (curve 2) [27]. At the ferroelastic phase transition temperature $T_{C}$ = 202 K, both curves show a kink in agreement with Refs. [17,18,19], which is due to the spin–phonon interaction. Above $T_{C}$, the phonon energy slightly decreases, in agreement with Refs. [4,17]. It must be noted that, at low temperatures, the anharmonic spin–phonon interaction plays an important role, whereas above $T_{C}$, there remain only the anharmonic phonon–phonon interactions. We have calculated the phonon energy for different relation $|J_{2}/J_{1}|$ values. $\omega_{0}$, and the phase transition temperature $T_{C}$ at which the kink appears, increase with an increasing $|J_{2}/J_{1}|$, i.e., with an increase in the magnetization. This shows the influence of the magnetic exchange interaction constants on the phonon energy, and the existence of a strong spin–phonon interaction. Our results are in good qualitative agreement with the experimental data of Kaczmarski and Wiesner [17]. It must be noted that an increase in $T_{C}$ with increases in the $|J_{2}/J_{1}|$ values was reported by Arnalds et al. [10], where the authors have theoretically studied the temperature dependence of the magnetization in an hcp Ising model. Unfortunately, we have not observed the two additional transitions at $T_{1}$≈ 180 K and $T_{2}$≈ 100 K [17]. Therefore, in our next paper, we will additively consider the temperature dependence of the dielectric constant, so as to obtain a better understanding of the structural changes in LCS. It must be noted that the phonon energy ω and the phase transition temperature $T_{C}$ (see Figure 3) decrease with a decrease in the NP size.

### 3.2. Temperature and Size Dependence of the Damping of the A_*1*g_ Phonon Modes $\omega_{0}$ = 32 and 72 cm*^−1^* in Bulk LCS

**Figure 2:**
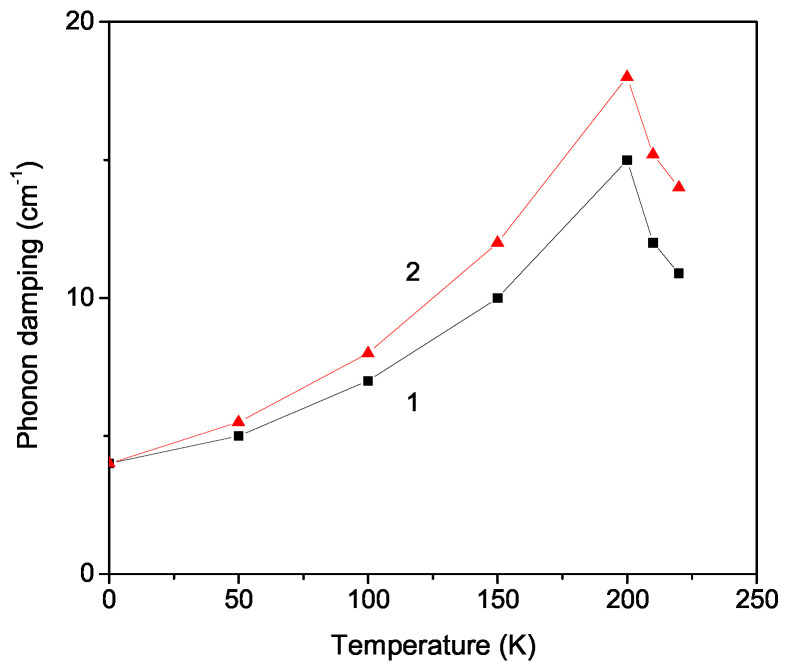
Temperature dependence of the phonon damping γ in bulk LCS for two phonon modes $\omega_{0}$ = 32 cm^−1^, R>0 (1), and 72 cm^−1^, R<0 (2), with $|J_{2}/J_{1}|$ = 0.3.

Figure 2 demonstrates the temperature dependence of the phonon damping γ for both phonon modes, $\omega_{0}$ = 32 cm^−1^ (curve 1) and $\omega_{0}$ = 72 cm^−1^ (curve 2), with a fixed ratio $|J_{2}/J_{1}|$ = 0.3. It can be seen that both damping curves increase with an increase in temperature *T*, for both cases R>0 and R<0, because γ is proportional to $R^{2}$. This means that the Raman peaks are broader by higher temperatures. Let us emphasize that the experimentally obtained broadened peaks in the Raman spectra of NPs, and especially of LCS NPs, cannot be understood within the random phase approximation (RPA) for small particles. We go beyond the RPA, taking into account all correlation functions, using the method of Tserkovnikov [26], and calculate the phonon damping effects in LCS NPs, including anharmonic spin–phonon and phonon–phonon interactions. At the phase transition temperature $T_{C}$, there is again a kink. Above $T_{C}$, the damping begins to decrease because the anharmonic spin–phonon contribution vanishes, and there remain only the anharmonic phonon–phonon interactions. A similar experimental behavior for the full width at half-maximum (FWHM), which corresponds in our model to the phonon damping for the second mode $\omega_{0}$ = 72 cm^−1^, is observed by Kaczmarski and Wiesner [17].

### 3.3. Size Dependence of the Ferroelastic Phase Transition Temperature $T_{C}$

**Figure 3:**
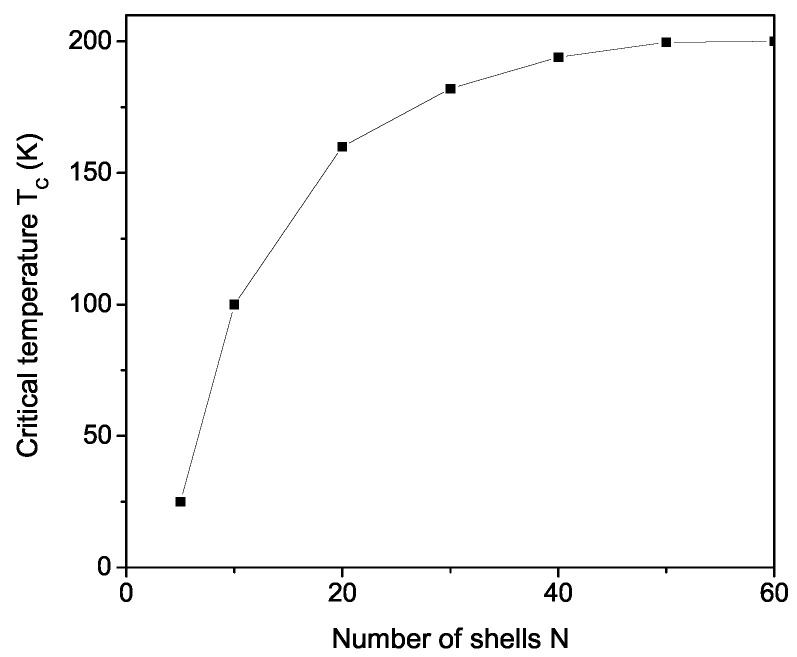
Size dependence of the ferroelastic phase transition temperature $T_{C}$ in LCS; *N* is the number of NP shells.

We have also calculated the size effects of the ferroelastic phase transition temperature $T_{C}$ from the shift of the kink in the temperature dependence of the phonon energy ω(T). To delineate this, we define a NP with a cubo-octahedral shape, centering the origin at a specific spin within the particle, and encompassing all other spins within shells. These shells are delineated by n=1,⋯,N, where n=1 designates the central spin and n=N corresponds to the surface shell of the system. The exchange interaction $J_{ij}\equiv J\left(r_{i}-r_{j}\right)$ relies on the distance between spins, inversely proportional to the lattice parameters. Surface effects are factored in by employing distinct coupling parameters within the surface layer (n=1 or *N*), denoted as $J_{s}$, compared to the bulk parameter $J_{b}$. This enables a microscopic-level discussion of the properties. We use the relation of the smaller interaction constant on the surface $J_{s}$ compared to that in the bulk $J_{b}$, i.e., $J_{s}<J_{b}$. It can be seen that $T_{c}$ decreases with decreases in the NP size and the number of NP shells *N* (see Figure 3), underlining the significance of NP size effects in modulating the ferroelastic behavior of materials. Our result is in coincidence with the experimental data of Borisov et al. [21], who have found, from critical anomalies in the velocity of shear ultrasound, that the transition in LCS NPs was shifted to low temperatures by about 6 K, compared to that in the bulk LCS. Let us note that this is not the case in all compounds. For example, for BaTiO_3_ and PbTiO_3_, ferromagnetic NPs $T_{C}$ also decrease with a decrease in NP size *d* but, in MnO for example, BiFeO_3_ or other antiferromagnetic NPs the Neel temperature increase with a decrease in *d* [28,29]. It depends on the strain that appears in the compound by changing the size.

### 3.4. Temperature Dependence of the Specific Heat $C_{p}$ in Bulk LCS

**Figure 4:**
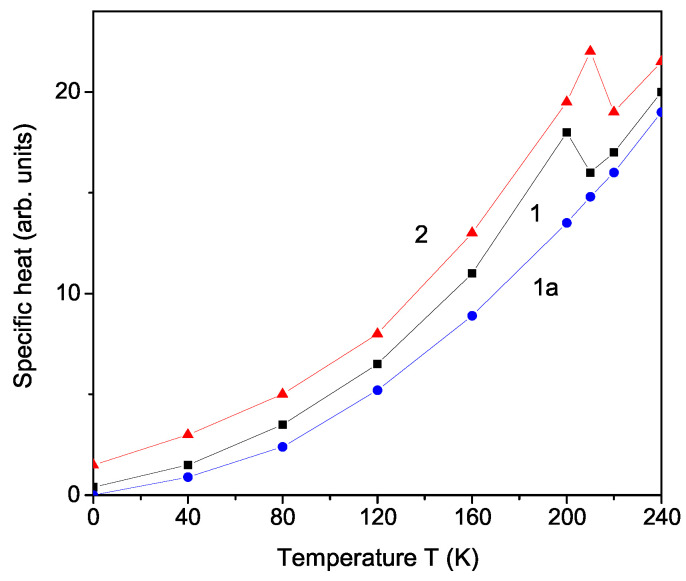
Temperature dependence of the specific heat $C_{p}$ in (1) pure bulk LCS and (2) K-doped bulk LCS, *x* = 0.15; (1a) a LCS NP with *N* = 5 shells.

Figure 4 shows the temperature dependence of the specific heat $C_{p}$ in bulk LCS calculated from the equation $C_{p}=d\langle H\rangle /dT$. It can be seen that, at $T_{C}$∼ 202 K, a peak appears, which is due to the spin–phonon interaction *R* (see Figure 4, curve 1). Let us note that, for *R* = 0, this anomaly disappears. Unfortunately, there do not exist experimental data for $C_{p}\left(T\right)$ in LCS around $T_{c}$. Delfino et al. [30] have investigated $C_{p}\left(T\right)$ in LCS in the temperature interval 300 K ≤ T ≤ 520 K, where no phase transitions are detected. Furthermore, it is noteworthy to highlight previous ndings reporting the presence of a discernible kink in Cp at TC in analogous compounds such as LiNH_4_SO_4_, LiKSO_4_, and Ru-doped LiKSO_4_, as meticulously documented by Polomska et al. [31], Kassem et al. [32], and Yurtseven et al. [33], respectively. A noticeable trend is the reduction in Cp with diminishing NP size, where the peak at TC diminishes in magnitude and shifts towards lower temperature values. This effect is accentuated in very small NPs, ultimately leading to the disappearance of the peak, as illustrated in Figure 4, curve 1a. Unfortunately, experimental data for Cp(d) in LCS are not available.

### 3.5. Ion Doping Dependence of the Phase Transition Temperature $T_{C}$ and the Specific Heat $C_{p}$

**Figure 5:**
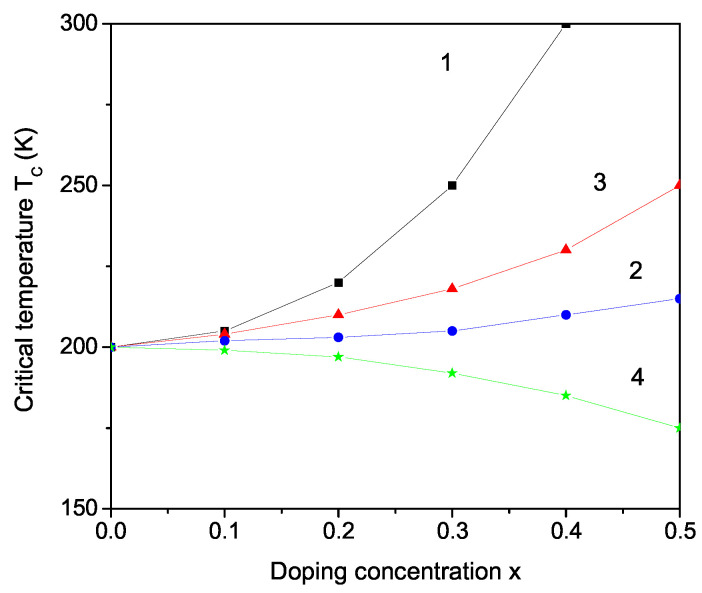
The phase transition temperature $T_{C}$ in bulk LCS as a function of different ion doping concentration *x* at the Cs site: (1) K; (2) NH_4_; (3) Rb; and at the Li site (4) K.

Finally, we will discuss the doping effects on the phase transition temperature $T_{C}$ and the specific heat $C_{p}$ on the microscopic level, for example by replacing the Cs ion (1.81 Ȧ) with the smaller K ion (1.52 Ȧ). A compressive strain appears, i.e., the exchange interaction constant at the doped states is larger than that of the undoped states, $J_{d}>J_{b}$. This leads to an increase in $T_{C}$ with an increase in the K ion doping concentration *x*. The result is shown in Figure 5, curve 1. It must be noted that $C_{p}$ also increases with an increase in K dopant *x* (see Figure 4, curve 2). As a similar behavior, we obtain an increase in $T_{C}$ by substituting the Cs^+^ ion with N$H_{4}^{+}$ ion (see Figure 5, curve 2), which is in agreement with the experimental data of Czaja [22]. Curve 3 in Figure 5 presents the increase in $T_{C}$ in LCS after doping with Rb^+^ ions, in coincidence with the result of Zapart et al. [23]. It must be noted that the $T_{C}$ of LiKSO_4_ is 708 K [33], whereas of LiRbSO_4_, it is 477 K [34]. We would also observe an increase in $T_{C}$ and $C_{p}$ by doping with Sm^3+^ or Dy^3+^ ions on the Cs site, as reported by Kassem et al. [35], when doped with the last two ions LiRbSO_4_. Tuszynski et al. [13] and Melo et al. [4] reported an increase in $T_{C}$ as a function of an applied uniaxial stress.

Furthermore, substituting the Li^+^ ion with Na^+^, K^+^ or Rb^+^ ions, which is characterized by larger ionic radii (0.97, 1.33, and 1.47 Ȧ, respectively), compared to the host Li ion (0.9 Ȧ), induces a tensile strain [36]. This means that we must choose the relation $J_{d}<J_{b}$ that would lead to reduction in the phase transition temperature $T_{C}$ compared to that of pure LCS. Thus, our model can explain the dependence of $T_{C}\left(x\right)$ on a microscopic level. Unfortunately, there are no experimental data for this behavior.

## 4. Conclusions

In conclusion, the phonon energy and damping of the A_1*g*_ modes $\omega_{0}$ = 32 cm^−1^ and 72 cm^−1^ are calculated. It is observed that both modes have different temperature dependences. The first mode increases, whereas the second one decreases with an increase in temperature *T*. This behavior is explained with the different sign of the anharmonic spin–phonon interaction constant *R*. At the ferroelastic phase transition temperature $T_{C}$∼202 K is a kink in both curves, due to a strong spin–phonon interaction in LCS. Above $T_{C}$, the phonon energies slightly decrease. The influence of the exchange interaction constants *J* on the phonon modes is shown. The phonon modes increase with an increase in the $|J_{2}/J_{1}|$-value. The phonon damping for both phonon modes increases with the temperature *T*, and shows a kink at $T_{C}$. The contribution of the anharmonic spin–phonon and phonon–phonon interactions in different temperature intervals is discussed. $T_{C}$ decreases with a decrease in NP size. Substituting the Cs ion with K, NH_4_, or Rb ions enhances the $T_{C}$, whereas replacing the Li ion with Na, K or Rb reduces the $T_{C}$. The specific heat $C_{p}$ increases with increases in temperature and K ion doping concentration, and shows a kink at $T_{C}$. $C_{p}$ is reduced in LCS NPs compared to the bulk case.

We hope that our investigation will result in other experimental and theoretical studies of the properties of bulk and nanostructured, pure and doped LCS compounds.

## Data Availability

Data are contained within the article.
